# Feasibility, acceptability, and user perspective of a next-generation wrist-worn receiver and digital pills for HIV pre-exposure prophylaxis: a pilot study

**DOI:** 10.3389/fpsyt.2026.1550058

**Published:** 2026-05-15

**Authors:** Dhruv Nimbalkar, G. R. Goodman, J. Hokayem, H. Albrechta, L. Loo, J. S. Lee, C. E. Goldfine, C. O’Cleirigh, K. Mayer, P. Alpert, C. Carnes, P. R. Chai

**Affiliations:** 1 The Fenway Institute, Fenway Health, Boston, MA, United States; 2 Boston University School of Public Health, Boston, MA, United States; 3 Department of Emergency Medicine, Brigham and Women’s Hospital, Boston, MA, United States; 4 Department of Psychiatry, Massachusetts General Hospital, Boston, MA, United States; 5 Department of Medicine, Beth Israel Deaconess Medical Center, Boston, MA, United States; 6 EtectRx, Gainesville, FL, United States; 7 Department of Psychosocial Oncology and Palliative Care, Dana Farber Cancer Institute, Boston, MA, United States; 8 The Koch Institute for Integrated Cancer Research, Massachusetts Institute of Technology, Boston, MA, United States

**Keywords:** digital pill, digital pill system, HIV prevention, HIV prevention (PrEP), ingestible electronics, ingestible sensors, medication adherence (MeSH)

## Abstract

**Objective:**

The objective of this study was to assess the feasibility and acceptability of the next-generation wrist-worn receiver (“Reader”) coupled with the ID-Cap digital pill system (DPS) to measure adherence to oral HIV PrEP (pre-exposure prophylaxis) via an ingestible radiofrequency sensor.

**Methods:**

This prospective cohort study enrolled 15 participants who were prescribed oral tenofovir diphosphate/emtricitabine (TDF/FTC) as PrEP. Participants were asked to ingest one digital pill daily for 30 days. Adherence was also measured using pill counts at the 30-day follow-up visit, which was compared to DPS-recorded ingestions (including both system-detected and manually annotated ingestions). The primary outcomes were system feasibility, defined as the ability of participants to engage with and operate the DPS throughout the 30-day study period and acceptability, as reported via the System Usability Scale (SUS). Qualitative interviews were conducted to receive feedback from the participants. The secondary outcome was the accuracy of the DPS, measured via a Pearson correlation comparing system-detected ingestions with pill count at 30-day follow-up.

**Results:**

14 participants consistently recorded ingestions using the DPS over the 30-day study period, demonstrating feasibility of the system. A technical error occurred on one participant’s wrist-worn Reader that rendered it unable to detect ingestions. Weekly engagement remained consistent across the study period. N = 12 participants returned pills at the final study visit. Based on pill count, 343 doses were ingested overall; 278 doses (81%) were detected by the DPS. DPS-based detection was strongly correlated with pill counts (r=0.75, p=0.0047). Participants had a mean SUS score of 78.0 (± 9.4), indicating good acceptability of the system. Qualitative feedback demonstrated good usability and convenience of operating the DPS. For N = 6 participants who had previously used a lanyard-based DPS Reader, all preferred the new wrist-worn system.

**Conclusion:**

A wrist-worn ingestible sensor system is acceptable and feasible among individuals to record PrEP adherence. The system is accurate in detecting ingestion events, in comparison to more traditional measures of adherence, and individuals can maintain engagement with the system over time. These findings warrant further evaluation in larger studies but strongly suggest that wrist-based form factors are suitable for integration into DPS technology.

## Introduction

Although efficacious options for pharmacotherapy to prevent HIV infection exist, the HIV epidemic continues to cause significant morbidity and mortality globally. As a strategy to End the HIV Epidemic (EHE), maximizing adherence to oral HIV pre-exposure chemoprophylaxis (PrEP) has the potential to minimize new HIV infections (1). Despite the efficacy of oral PrEP, adherence remains suboptimal, especially among key populations like individuals with substance use disorders (SUD) and men who have sex with men (MSM) (2, 3). For some individuals, the introduction of injectable cabotegravir and lenacapravir may address adherence challenges, but implementation and infrastructure to support cabotegravir is still nascent, and lenacapravir is not yet approved by regulatory authorities (4, 5). Additionally, operational challenges in the provision of injectable PrEP to individuals facing health disparities, including substance use, remain unresolved. There is therefore a continued need to support oral PrEP adherence in key populations at high risk of HIV infection.

Many different strategies exist to measure and provide feedback surrounding PrEP adherence (6). These adherence strategies are categorized into indirect methods (which infer medication ingestion) and direct methods (which confirm ingestion) (7). Each strategy has unique features that make it suitable for different individuals, but also comes with its own drawbacks. For example, while self-reported adherence is simple and easy to implement clinically, it is prone to bias. In contrast, measuring the concentration of PrEP in the blood directly allows for direct confirmation of PrEP ingestion, but lacks contextual information about day-to-day adherence patterns, which may be useful for informing behavioral interventions to support PrEP adherence (6, 8).

**Figure 1:**
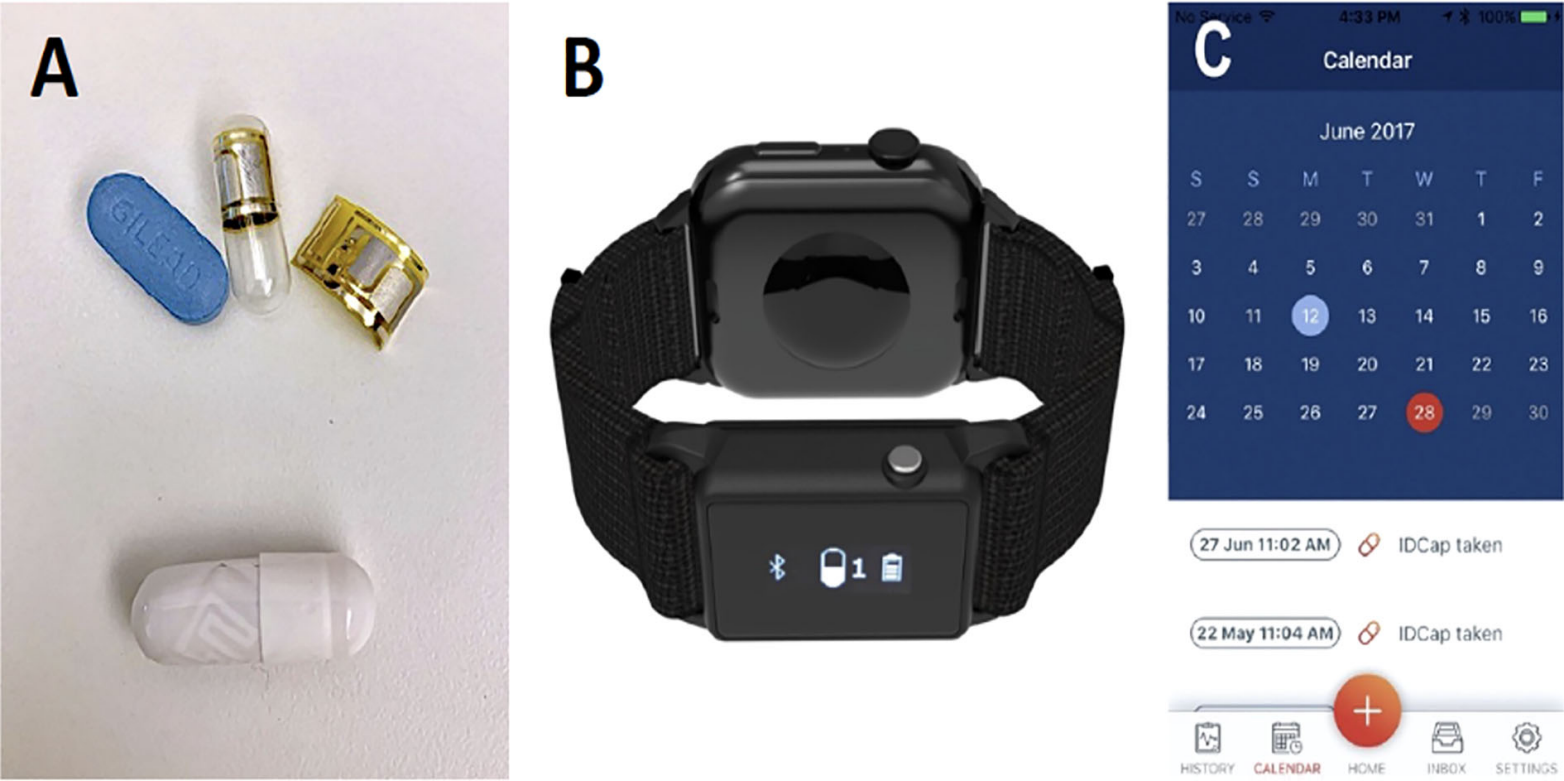
The DPS (ID-Cap System, etectRx) comprises three parts: the digital pill **(A)**, which, following ingestion, sends a signal upon activation in the stomach to a reader device worn on the wrist that has the option to be integrated onto a smartwatch band as shown above **(B)**, which stores and relays ingestion data to a participant-facing smartphone application **(C)**.

**Figure 2:**
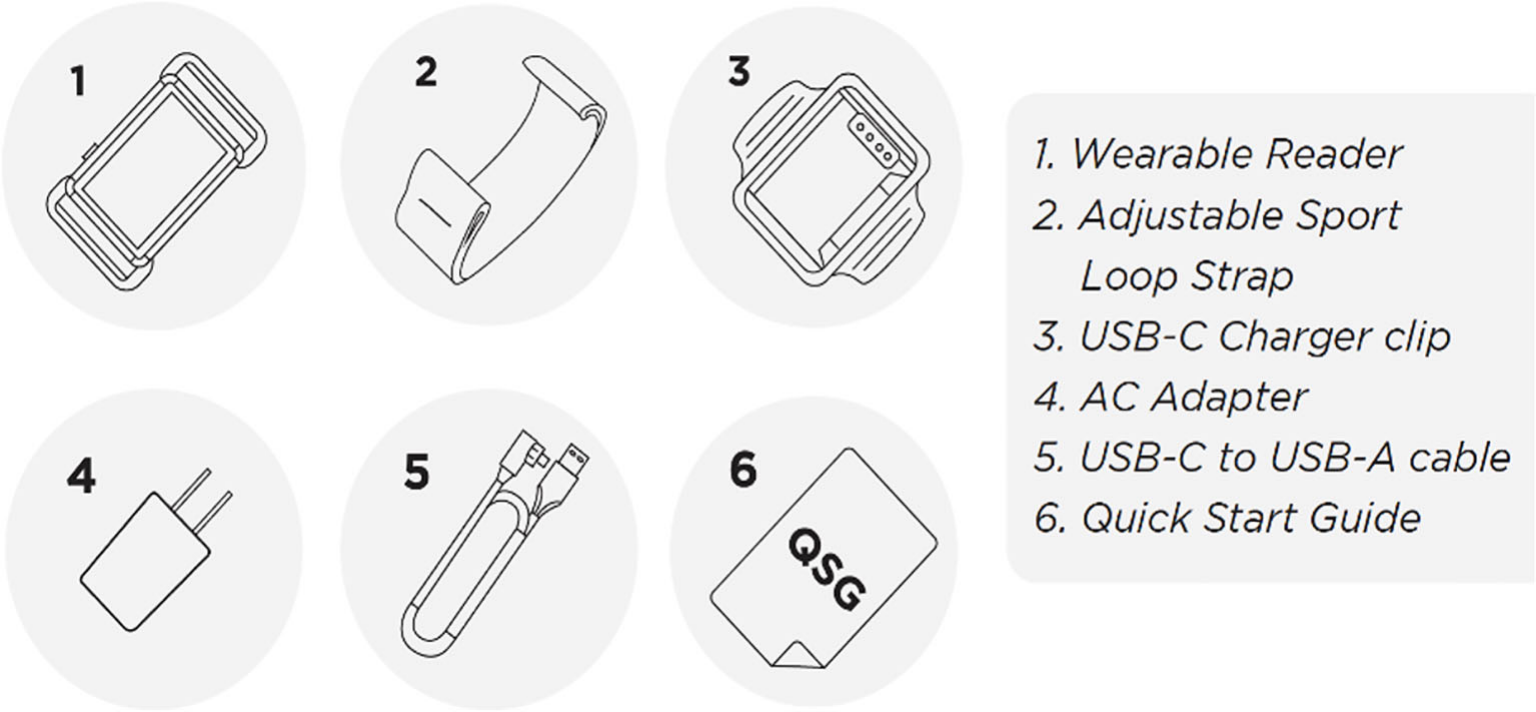
The contents of the DPS package provided to participants (ID-Cap System, etectRx) comprises of: the wearable Reader (1), the adjustable sport loop strap (2), the Reader charging clip (3), the charging adapter (4), the charging cord (5), and a quick start training guide (6).

One unique strategy to measure PrEP adherence is through the use of a digital pill system (DPS) (9) (**Figure 1**). The DPS utilizes standard gelatin capsules with integrated radio frequency sensors that over-encapsulate PrEP, facilitating the “digitization” of medications such as PrEP. The overencapsulated, gelatin capsule coated PrEP increases the size of the pill less than 1 millimeter. Once ingested, gastric chloride ions activate the radiofrequency sensor within the “digital pill,” which projects a signal off the body that is acquired by a wearable Reader device (10) (**Figure 2**). Subsequently, the Reader communicates ingestion data to a smartphone app and online dashboard, allowing for real-time monitoring by both patients and research teams, as well as contextualization of PrEP adherence and nonadherence events (11). Nonresorbable components of the digital pill (radiofrequency sensor) are excreted in the stool.

Digital pills have previously been used to measure adherence to a variety of medication regimens, including antidiabetic agents, antihypertensives, heart failure therapies, opioids for pain management, transplant medications, and antiretroviral agents (9, 12–17). Despite successes in measuring medication adherence in these contexts, qualitative work has identified the use of a wearable Reader (traditionally worn on a lanyard around the neck during medication ingestions) as both a facilitator of and a barrier to use of DPS technology. Key feedback from end users has highlighted the need to develop a smaller, less obtrusive system to improve overall perceived usability and overall adoption of the DPS for adherence measurement (18). This paper describes the development of a miniaturized, wrist-worn Reader system as part of an open-label clinical trial that aimed to explore the feasibility and acceptability of this next-generation Reader to measure adherence to PrEP digital pills.

## Methods

### Sensor development and testing

Based on previous user feedback of the DPS, we sought to miniaturize the microchip within the existing lanyard based reader to prepare it for integration into a wirst-based Reader. Reducing the size of the existing reader was achieved by reducing the existing printed circuit board reader into a microchip. The digital interface to the IC was validated via computer interface to ensure proper IC communication. Internal registers were programmed and then read out again to assure bit correlation. Several IC test modes were utilized during IC validation to verify the individual main networks, including the quadrature local oscillator (QLO), the direct down conversion sampled-data receiver (DDSD), the proprietary burst detect network (BDN) and the power management block. The BDN was then evaluated for signal magnitude precision, dynamic threshold control, noise and impulse rejection capability, and Receive Signal Strength conversion accuracy. After successful chip validation, the new chip was integrated into a prototype form factor to facilitate final verification and validation of the miniaturized reader. Off the shelf materials were used to fashion the wristband and other components of the system. A finalized wrist-borne Reader was tested in laboratory conditions prior to integration into the current study (19). No modifications were required for the ingestible sensor component of the system.

#### Participants

We enrolled 15 adult participants ≥18 years old who were HIV-negative, already prescribed oral TDF/FTC as PrEP for at least 30 days, owned a smartphone (Android or iOS), and had qualifying laboratory work (creatinine clearance and liver functions tests) demonstrating that it was safe to maintain them on TDF/FTC. We excluded individuals with a history of inflammatory bowel disease (Crohn’s or ulcerative colitis), significant prior gastrointestinal surgery, or gastrointestinal neoplasms, and those with known allergies to zinc and silver (components of the digital pill printed circuit board). Previous pilot trials of early stage technologies have enrolled sample sizes of 10–15 individuals to measure feasibility and acceptability (20). Given this, we sought to enroll N = 15 individuals in this study.

#### Procedures

Participants were recruited at Fenway Health, a federally qualified health center based in Boston, Massachusetts with expertise in caring for LGBTQ+ individuals. Individuals were also recruited through community-based outreach in the Boston metropolitan area and queries of the electronic health record at Fenway. Recruitment efforts were conducted between February 2023 and June 2024. All study procedures were approved by the Fenway Community Health Institutional Review Board (IRB).

Interested individuals were pre-screened and those meeting preliminary eligibility criteria attended an in-person Screening Visit (Visit 1), where written informed consent was obtained and laboratory work confirming PrEP eligibility was reviewed or conducted onsite as needed. Following confirmation of eligibility, participants attended an Enrollment Visit (Visit 2), where they completed a baseline quantitative assessment and received an adapted version of a standardized training program on operation of the DPS, including an overview of each component and a demonstration of how to use the system. Participants were introduced to the next-generation, wrist-worn Reader device (which replaced the previous lanyard-worn Reader), which was integrated into an adjustable sport loop wristband. They were also oriented to the companion smartphone app, where they could view their PrEP ingestion history. Participants had the ability to manually annotate ingestion events via the app; they were instructed to do so only in the event that they: (1) ingested a digital pill, but the ingestion event was not displayed in the app, or (2) ingested a digital pill, but did not use the Reader at all. At the end of the Enrollment Visit (Visit 2), participants were dispensed a 30-day supply of digital PrEP pills (i.e., over-encapsulated Emtricitabine/Tenofovir) and instructed to take one digital pill per day throughout the study period.

At the end of the 30-day study period, participants returned for a final Month 1 Visit (Visit 3), where the study team collected all DPS equipment, counted any unused digital pills, and conducted a timeline follow back discussion to better understand the context of any DPS-detected nonadherence events during the prior 30 days (i.e., any 24-hour period in which no ingestion was registered on the ID-Cap System). A semi-structured qualitative interview was conducted by a trained member of the study team to explore participants’ experiences using the next-generation Reader. Participants also completed the System Usability Scale (SUS) as a quantitative measure of perceived system acceptability (21). Remuneration was provided following completion of procedures at all three study visits. Study procedures were conducted between January 2023 and July 2024.

### Measures

#### Quantitative assessment

On the baseline quantitative assessment, completed at the Enrollment Visit (Visit 2), participants self-reported sociodemographics (e.g., age, race, ethnicity, sexual orientation, relationship status, education, income), PrEP use (e.g., duration of PrEP use, self-reported missed doses during prior month) and HIV testing history (e.g., time since last HIV test, reason for last HIV test), and sexual history (e.g., number of recent sexual partners, condom use during prior three months). The assessment also measured depression (Patient Health Questionnaire 8-item; PHQ-8) (22), anxiety (Generalized Anxiety Disorder 7-item; GAD-7) (23), baseline attitudes towards technology use (16-item Media Technology Usage and Attitudes Scale; MTUAS) (24), medical mistrust (6-item adapted Group-Based Medical Mistrust Scale; GBMMS) (25), and substance use history (2-item Alcohol, Smoking and Substance Involvement Screening Test; ASSIST) (26). Data was stored and managed in the Research Electronic Data Capture (REDCap) system.

#### Digital pill system usage

We measured the feasibility of the system through engagement and use of the DPS. Data was queried on a weekly basis and adherence events were defined as either (1) system-detected (i.e., ingestion of a digital pill capsule and acquisition of this signal on the Reader) or (2) manually-annotated (i.e., participant-initiated recording of an ingestion using the app in the event of perceived nonfunction of the DPS). Potential discrepancies in data (e.g., a system-detected and manually annotated ingestion within seconds of each other) were documented and reviewed with participants during the timeline follow-back discussion at the Month 1 Visit (Visit 3). These discrepancies were reconciled via participant self-report, analysis of Reader data, and discussion among the study team.

#### Pill counts

At the Month 1 Visit (Visit 3), participants were asked to return the original dispensed pill bottle, including any unused medication from the study period. Leftover pills were counted; the difference between the number of dispensed pills (30) and the remaining pills served as the number of expected ingestions for each participant over the study period.

#### System usability scale

At the Month 1 Visit (Visit 3), following 30 days of operating the next-generation DPS, participants completed the SUS, a validated 10-item measure to evaluate the acceptability and perceived usability of a technological system (i.e., the wrist-based Reader) (21).

#### Qualitative interview

Qualitative data was collected via individual exit interviews conducted at the Month 1 Visit (Visit 3). Interviews followed a semi-structured qualitative interview guide, grounded in the Technology Acceptance Model, a conceptual model that explores the perceived usefulness, intended operation and actual real-world adoption of technologies (27). We have previously leveraged this conceptual model to obtain qualitative data surrounding real-world operation of the DPS (18, 28). Interview themes included an exploration of participants’ experiences with the next-generation DPS, overall experiences using the system, experiences operating the wrist-worn Reader, suggested improvements and recommendations to further optimize the system design, and privacy-related considerations related to system use (**Table 1**). For participants who had previous utilized the DPS, we asked them to describe the experience using a wrist-worn Reader compared to previous versions of the system.

**Table 1:** Sample interview content areas & questions.

Content area	Sample questions and probes
Overall experiences with DPS	• Overall, how was your experience using the DPS? • How easy or difficult was it to understand and operate? • Could you walk us through a typical day using the DPS?
Experiences with operation of wrist-worn Reader	• What did you like/dislike about the wrist-worn Reader? • What helped you to use the wrist-worn Reader each day? • What got in the way of using the wrist-worn Reader each day?
Suggested improvements and recommendations	• What improvements would you make to the wrist-worn Reader? • What would be the best way to integrate the DPS into your PrEP care? • Who do you think would benefit most from using the DPS? Why?
Privacy considerations related to DPS use	• Overall, how did you feel about your personal privacy while using the DPS during this study? • Did you worry that we were able to track whether you took your medication each day? • Who should have access to adherence data from the DPS?

### Analyses

#### Baseline demographic and quantitative assessment data

Descriptive statistics were calculated to categorize sample demographics (**Table 1**). Mean scores were calculated for depression (PHQ-8), anxiety (GAD-7), baseline attitudes towards technology use (MTUAS), and medical mistrust (GBMMS) (**Table 1**). For the 2-item measure of substance use history (ASSIST), frequency data for each item (i.e., lifetime use) are reported (**Table 2**).

**Table 2:** Baseline characteristics (N = 15).

Covariate	Subcategories	N(%)
Age, mean (sd)		40.4 (16.4)
Body mass index (BMI), mean (sd)		26.9 (4.0)
Sex at birth, N (%)	Male	15 (100)
	Male	14 (93.3)
	Non-binary/Gender fluid/Gender queer	1 (6.7)
Ethnicity, N (%)	Hispanic	3 (20.0)
	Not Hispanic	12 (80.0)
	Asian	1 (6.7)
	Black or African American	1 (6.7)
	White	11 (73.3)
	More than one race	2 (13.3)
Education level, N (%)	Some college (no degree) or less	1 (6.7)
	College degree	9 (60.0)
	Some Graduate work (no degree)	1 (6.7)
	Graduate/Professional degree	4 (26.6)
Annual income, N (%)	$12,000 to $17,999	1 (6.7)
	$18,000 to $23,999	2 (13.3)
	$24,000 to $29,999	1 (6.7)
	$30,000 to $59,999	1 (6.7)
	$60,000 and above	10 (66.6)
PrEP adherence, N (%)	≤ 4 days out of 7 days per week	1 (6.67)
	≥ 5 days out of 7 days per week	14 (93.33)
Baseline depression, anxiety, attitudes toward technology, and medical mistrust (N = 15)		
PHQ-8 score, mean (sd)	2.8 (2.2)	
GAD-7 score, mean (sd)	3.7 (3.6)	
MTUAS score, mean (sd)	55.9 (8.2)	
GBMMS score, mean (sd)	1.8 (0.6)	
Baseline substance use history, per ASSIST (N = 15)		
Substance	Lifetime use, N (%)	
Tobacco	8 (53.33)	
Alcohol	13 (86.67)	
Cannabis	12 (80)	
Cocaine	3 (20)	
Amphetamine	4 (26.67)	
Inhalants	6 (40)	
Sedatives or sleeping pills	3 (20)	
Hallucinogens	6 (40)	
Opioids	2 (13.33)	
Sexual history (past 3 months) [N = 15]		
Variable		
Sexual activity, N (%)	Yes	14 (93.33)
	No	1 (6.67)
Substance use with sex, N (%)	Never	3 (21.43)
	Almost Never	2 (14.29)
	Sometimes	6 (42.86)
	Almost every time	2 (14.29)
	Every time	1 (7.14)
Condom use. N (%)	Never	4 (28.57)
	Almost Never	6 (42.86)
	Sometimes	2 (14.29)
	Almost every time	2 (14.29)
STI history, N (%)	Yes	2 (13.33)
	No	13 (86.67)
Number of sexual partners [Median (IQR)]	3.5 (3-4)	

PHQ-8, Patient Health Questionnaire; GAD-7, Generalized Anxiety Disorder; MTUAS, Media Technology Usage and Attitudes Scale; GBMMS, Group-Based Medical Mistrust Scale; ASSIST, Alcohol, Smoking and Substance Involvement Screening Test.

#### Primary outcome: feasibility of DPS with wrist-worn reader

Feasibility was defined as the ability of participants to operate the DPS throughout the 30-day study period. The DPS interface was queried for the number of both system-detected and manually annotated ingestions over the study period. We assumed feasibility of using the DPS to measure adherence if participants were able to record ingestion events using the DPS at least once per week during the study period. Engagement with the DPS was assessed as the number of system-detected and manually annotated PrEP ingestions by week and overall at the end of the study period.

#### Primary outcome: acceptability of DPS with wrist-worn reader

Acceptability was measured quantitatively via the SUS. Scores of at least 68 were considered acceptable, as has been established as a benchmark for acceptability on this measure (21). Quantitative data was supplemented with qualitative interviews to understand the operation of the DPS and the acceptability of use during the study period. One participant, who was unable to operate the Reader due to a known technical error during the study period, was excluded from this analysis.

#### Secondary outcome: accuracy of DPS with wrist-worn reader and engagement with the DPS over the study period

Raw adherence data was obtained from the DPS interface over the 30-day study period for all participants. Three participants were excluded from this analysis (two who did not return pill bottles for pill counts after 30 days, and one who experienced a technical error in the Reader, rendering it inoperable during a significant portion of the study period). We derived a composite value of expected ingestions by subtracting the number of leftover pills counted at the final study visit from 30. We compared this value to adherence data provided from the DPS during the same 30-day study period. Next, we calculated a Pearsons’ correlation coefficient to compare all adherence data from the DPS to pill counts conducted at 30 days. We defined engagement as consistent use of the DPS over the study period in addition to a strong positive correlation between expected ingestions (pill count) and DPS recorded adherence events.

#### Qualitative data

A rapid qualitative analysis was conducted using a framework matrix to understand user responses to and experiences with the next-generation DPS. Interviews were professionally transcribed and transcripts were read by two trained members of the study team, who organized data into a framework matrix format and extracted preliminary themes (29). The matrix was reviewed by the full study team and salient themes were finalized. Emergent themes were discussed among the study team and added to the framework matrix. Any discrepancies in data organization and analysis were adjudicated by the study team.

## Results

### Enrollment and demographics

Over the study period, 23 individuals were pre-screened, of whom 21 met preliminary eligibility criteria (91.3%). The only reason for ineligibility was not being on PrEP (n=2). Of those eligible at pre-screening, 15 attended a formal Screening Visit (Visit 1; 71.4%), and all enrolled in the study. Six of these participants had previously participated in another study that used the same DPS, but had utilized a wearable Reader with a lanyard that was intended to be worn around the neck. All participants completed all study visits.

Mean age was 40.4 years (SD 16.4) (**Table 2**). All participants were assigned male at birth. Participants were predominantly White (73.3%), followed by mixed race (13.3%), Asian (6.7%) and Black or African American (6.7%). The majority identified as non-Hispanic (80%). Most participants (60%) had a college degree, and 66.6% reported an annual income of $60,000 or above. Mean body mass index (BMI) was 26.9.

### Baseline characteristics

At baseline, participants had low depression and anxiety scores (PHQ-8: M = 2.8 [SD 2.2]; GAD-7: M = 3.7 [SD 3.6]), and were generally receptive of the use of technologies on the MTUAS (M = 55.9, SD = 8.2). Participants were also trustful of the medical system per the GBMMS (M = 1.8, SD 0.6). All participants endorsed substance use, with the majority reporting alcohol (86%) and cannabis use (80%). Nearly all participants self-reported prevention-effective PrEP adherence over the past three months (at least 4 doses of PrEP taken per week), and 73% (N = 11) reported daily PrEP adherence during the same timeframe. Almost all participants (93%, N = 14) reported being sexually active over the past three months, with a median of 3.5 reported partners. Over half the sample (N = 9, 60%) reported the use of substances during sexual activity. Additionally, 66% (N = 10) of participants reported seldom or never using condoms during intercourse. Only two participants (13%) reported a previous sexually transmitted infection (STI) in the previous three months.

### Feasibility

**Figure 3:**
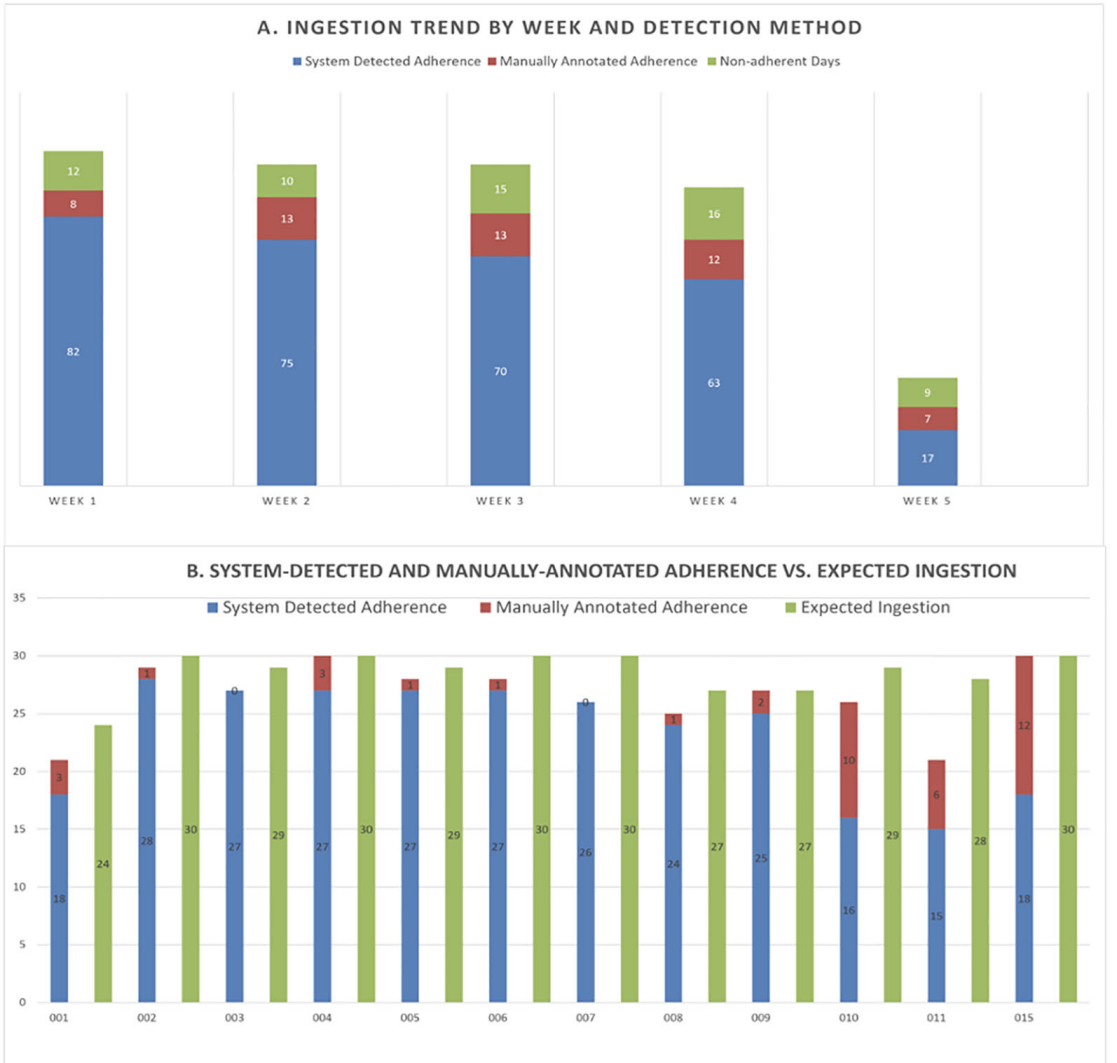
**(a)** Ingestion trends by week, ahowing consistent operation of DPS (N = 14). **(b)** Overall DPS-Recorded Ingestions, Including System-Detected and Manually Annotated (N = 12).

Across the 14 participants analyzed for this outcome, 422 person-days of adherence were recorded (**Figures 3a, b**). One participant was eliminated from this analysis as we experienced a technical error within the Reader. This one participant was unable to record any digital pill ingestions during the study period. On query, we found a programming error within the participant’s Reader that was preventing it from acquiring the digital pill signal and transmitting it to the smartphone app. We did not experience this issue with any of the other wrist-borne Readers during the study. In these days, the DPS recorded a total of 360 ingestions. Of these, 307 ingestions were system-detected (i.e., digital pill ingested, Reader correctly operated, and adherence data captured on app and online dashboard), and 53 ingestions were manually annotated via the app. Engagement with the DPS remained consistent over the 30-day study period. While overall DPS-recorded adherence decreased over the study period, participants also became more nonadherent to PrEP – suggesting that, even with decreasing adherence, use of the DPS did not wane over time. All participants were able to consistently record ingestion events at least once weekly with the DPS throughout the study period.

### Acceptability

The mean SUS score at the Month 1 Visit (Visit 3) was 78.0 (SD 9.4), suggesting that participants perceived the wrist-worn Reader to be above-average in terms of usability (average score on SUS: 68).

Qualitative feedback from individual interviews confirmed this finding, with most participants describing the daily operation of the system as acceptable. Participants described being able to rapidly integrate the wrist-worn Reader into their daily lives. Importantly, participants did not report that the added step of wearing a wrist-worn device prior to taking their daily digital pill was a significant barrier to or departure from their normal routine. The translation of the Reader from a lanyard-based system to one worn on the wrist was viewed as acceptable. The small size of the Reader, and corresponding charger which clipped directly onto the device, were described as simple-to-use innovations that improved the overall acceptability of the system. Six participants had previously participated in other DPS-related studies that featured the legacy Reader worn around the neck on a lanyard. All of these participants described the wrist-worn system as more usable and acceptable, as compared to the lanyard-based system. An emerging theme surrounding the use of the wrist-borne reader was that participants viewed it as more discrete, easier to use and potentially a device they might wear all day if additional capabilities were integrated into the system. Additionally, the presence of a small screen that indicated ingestion events on the body of the wrist-worn Reader was perceived as a facilitator that helped improve participants’ experience operating the DPS. Participants did not describe any other discomfort with operating and using the DPS during the study period including excreting the nonresorbable components in stool.

### Accuracy/engagement

Across N = 12 participants, the total number of cumulative expected ingestions was 343, based on pill counts at the Month 1 Visit (**Table 3**). Of these 343 expected ingestions, 318 (93%) were recorded by the DPS. Of these DPS-recorded ingestions, 278 (81%) were system-detected, meaning they were recorded by participants correctly using the DPS system such that PrEP ingestions were detected by the wrist-worn Reader; a total of 40 ingestions were manually annotated in the app. All individuals consistently engaged with the system by recording adherence data at least weekly during the study period. Pearson’s correlation coefficient demonstrated a significant, strong association between DPS-recorded adherence events and pill counts 0.75 (p=0.0047). At the month 1 visit, participants reported various reasons for using the manual annotation. These ranged from participants forgetting to charge or wear the Reader, taking their digital PrEP pills in a location where they did not have access to the Reader or perceived Reader malfunction.

**Table 3:** Overall PrEP adherence data – system-detected versus expected ingestions per pill counts (N = 12).

System-detected adherence	Manually annotated adherence	Total DPS-recorded adherence	Expected ingestions from end of study pill count
278	40	318	343

## Discussion

Despite advances in biobehavioral interventions for HIV, the HIV epidemic continues to persist. A key aim of ending the HIV epidemic is improving strategies to measure adherence to HIV treatment and prevention pharmacotherapy (1). While there are many strategies to measure adherence, there remains no gold standard (6). Digital pills represent a potential direct measure of adherence that may help to drive context-aware, personalized interventions to augment adherence (9, 11, 13). While digital pills have been established as a feasible means of measuring adherence to both HIV treatment and prevention, a key barrier has been the use of a wearable Reader device, worn via a lanyard around the neck, to acquire ingestion data from digital pills (18). Previous qualitative work has identified the use of a wrist-worn Reader system as a potentially more acceptable strategy to miniaturize and improve the usability of the DPS (18, 19, 30). This investigation advances digital pill technology from legacy lanyard-based Reader systems to a wrist-worn device that is grounded in real-world user feedback. This study demonstrated that a wrist-based system is both feasible and acceptable to users, and is able to produce accurate adherence data among individuals using PrEP – thereby advancing research surrounding current systems to support digital pills as a HIV pharmacotherapy adherence measurement tool.

Providing a suite of wearable device options with the capacity to acquire signals from the digital pill has been consistently described by participants as an innovation that would improve overall system usability. Grounded in this user feedback, we were able to successfully miniaturize the electronics of the lanyard-based Reader and develop a wrist-worn Reader that was feasible to use and acceptable by participants in this study. Over the 30-day study period, participants were able to engage with and effectively operate the wrist-worn Reader. Continuous use of the system over the study period suggests that key operational components to using a wrist-worn Reader – including charging the system, maintaining connectivity to a smartphone, and remembering to use the Reader while taking medications – was viable on day-to-day basis. Preliminary qualitative data also suggests that the user experience of the wrist-worn Reader was acceptable, and that the device was easily integrated into participants’ existing pill-taking routines. Interestingly, among a cohort of individuals in this study who had previously participated in DPS studies involving the use of a lanyard-based Reader, the wrist-worn Reader was described as a more acceptable iteration of DPS technology. These findings provide support for the integration of a wrist-worn Reader into the DPS in order to continue increasing the usability of the system.

Congruent with previous investigations, we demonstrated that adherence data detected by the DPS via a wrist-worn Reader strongly correlates with pill counts, a well-established (albeit indirect) metric for measuring adherence (9). Despite the greater movement of the wrist-worn Reader and its increased distance from the RF-emitting digital pill after ingestion—compared to the lanyard-worn device—we did not observe any changes in pill detection. This consistency is partly attributed to the wrist-worn Reader’s improved signal acquisition power (19). Furthermore, we encountered no failures in pill detection, indicating that the design of the wrist-worn Reader preserves the accuracy of the earlier Reader system. While no gold standard yet exists for measuring medication adherence in HIV prevention, our findings suggest that ingestion data acquired from the wrist-worn Reader is accurate and can be reliably used to inform behavioral interventions – and potentially clinical decision-making – for PrEP prescribing. For example, individuals with self-reported challenges in PrEP adherence may be given the DPS as a strategy to help assess why adherence challenges occur, and facilitate conversations with clinical teams around how to maximize adherence. Additionally, for individuals who are initiating PrEP and have sufficiently high risk exposure to HIV, the DPS could be used to help teach adherence skills, especially for individuals who may have never been prescribed a daily medication before. In both scenarios, the DPS can provide objective adherence data that is available on-demand for counseling and self-management—a key advance of this system over other objective adherence tools (6, 7).

A key consideration to future implementation of the DPS is overall cost of the system. While we conducted this study under a funded research grant, it will be important to consider the costs associated with deployment of the DPS including potential pharmacy costs and costs of personnel that may need to monitor and respond to adherence data. While our previous work demonstrates that adherence monitoring may be accomplished with minimal increase in personnel time, especially with the use of artificial intelligence tools that facilitate autonomous feedback around adherence (11, 30), a formal cost effective analysis will need to be undertaken in the future.

Despite the important findings of this investigation, several limitations should be noted. The study was conducted at a single site with expertise in deploying the DPS and providing care across the HIV treatment and prevention continuum. Sites with different infrastructure or resources may have varied experiences with the DPS. The study sample was predominantly male, white, and reported low levels of medical mistrust. Individuals from racial and ethnic minority communities and those with health disparities and historical medical mistrust may experience different perspectives of the DPS. We compared DPS-acquired adherence reports to pill counts to measure accuracy, and since no gold standard exists for measuring daily adherence, comparisons with other adherence metrics may yield different results. This study did not capture perspectives on acceptability or feasibility from providers, which may be important for understanding the system’s broader applicability in real-world clinical settings. In qualitative interviews, several participants noted that while the system was generally easy to use and convenient, improved accuracy would enhance their experience by reducing the need to verify ingestion events or rely on manual annotations. When ingestions were manually annotated, there was no mechanism to determine whether this was due to a system error, user error (e.g., not wearing the device during ingestion), or both. Additionally, the small sample size and pilot nature of the study limit the generalizability of the findings; future research with a larger cohort is needed to strengthen and validate these preliminary results.

Overall, this investigation demonstrates, for the first time, that incorporating a wrist-worn wearable Reader into the DPS is feasible, acceptable, and has reliability comparable to other Reader versions within the DPS. The wrist-worn Reader offers a novel option, grounded in user preferences, that is suitable for individuals participating in research studies to adherence events, or for clinical use to measure adherence. This flexibility could enable future biobehavioral studies in HIV treatment and prevention science to offer participants a choice of Reader—lanyard or wrist-worn—that aligns best with each participant’s routine and lifestyle. Based on our qualitative data, providing such options would likely improve the system’s acceptability and functionality in clinical research. The wrist-worn system offers several key advantages. First, wrist-based devices, such as smart watches and fitness trackers, are widely accepted and increasingly common. Second, the design of the wrist-worn system enables integration with other wearables; for example, the wrist-worn Reader system could be integrated into a smart watchband itself. Future studies should explore the usability of these integrations and the potential for incorporating the Reader into other wearable devices, clothing, or systems like smartphones.

## Data Availability

The original contributions presented in the study are included in the article/**Supplementary Material**. Further inquiries can be directed to the corresponding author.
